# Aloperine: A Potent Modulator of Crucial Biological Mechanisms in Multiple Diseases

**DOI:** 10.3390/biomedicines10040905

**Published:** 2022-04-15

**Authors:** Muhammad Tahir, Sakhawat Ali, Wenting Zhang, Boqiang Lv, Wenge Qiu, Juan Wang

**Affiliations:** 1 Faculty of Environment and Life, Beijing University of Technology, Beijing 100022, China; m.tahir.qau@hotmail.com (M.T.); 17600398559@163.com (W.Z.); a591283794@163.com (B.L.); 2 Beijing Institute of Technology, Beijing 100124, China; dr.sakhawatsaleem@hotmail.com

**Keywords:** apoptosis, cell cycle, autophagy, PI3K/Akt, NF-κB, Nrf2, Ras

## Abstract

Aloperine is an alkaloid found in the seeds and leaves of the medicinal plant *Sophora alopecuroides* L. It has been used as herbal medicine in China for centuries due to its potent anti-inflammatory, antioxidant, antibacterial, and antiviral properties. Recently, aloperine has been widely investigated for its therapeutic activities. Aloperine is proven to be an effective therapeutic agent against many human pathological conditions, including cancer, viral diseases, and cardiovascular and inflammatory disorders. Aloperine is reported to exert therapeutic effects through triggering various biological processes, including cell cycle arrest, apoptosis, autophagy, suppressing cell migration, and invasion. It has also been found to be associated with the modulation of various signaling pathways in different diseases. In this review, we summarize the most recent knowledge on the modulatory effects of aloperine on various critical biological processes and signaling mechanisms, including the PI3K, Akt, NF-κB, Ras, and Nrf2 pathways. These data demonstrate that aloperine is a promising therapeutic candidate. Being a potent modulator of signaling mechanisms, aloperine can be employed in clinical settings to treat various human disorders in the future.

## 1. Introduction

For centuries herbal remedies have been employed in therapeutic practices. In recent times, many medicinal plants have been intensively investigated for better understanding of their mechanisms of action and discovery of novel bioactive compounds. *Sophora alopecuroides* of the *sophora genus* has remained one of the most popular medicinal plants in eastern Asian countries. It has been utilized to treat dysentery and inflammation [1]. More than 20 bioactive quinolizidine alkaloids have been isolated from *Sophora alopecuroides* plant [2]. These alkaloids have been categorized into various distinct structural groups: matrine-type, aloperinetype, and cytisine-type [3]. One of the most frequently isolated quinolizidine alkaloids from the *Sophora* plant is aloperine. The molecular formula of aloperine is C_15_H_24_N_2_ [4]. The investigation to discover its stereochemical structure shows that an octa-hydro quinoline ring partly covered by a quinolizidine ring constitutes its distinctive tetracyclic ring core. Identifying its stereo-chemical structure has enabled the synthesis of its derivatives for therapeutic purposes [5]. In 1992, the Chinese state food and drug administration (SFDA) approved the administration of *sophora* isolated alkaloids for treating cancer [6]. Aloperine has been widely investigated in a broad range of diseases. A literature review showed that aloperine could produce inflammation and tumor inhibitory effects [7,8]. It could also alleviate allergies and viral infections [9]. It is well known that most therapeutic agents produce beneficial effects by targeting signaling mechanisms. Investigations to understand the aloperine mediated remedial effects in different diseases revealed that aloperine could modulate various cellular signaling mechanisms to combat disease conditions.

Apoptosis or programmed cell death is a frequent mechanism of action of many drugs. Caspase-dependent and mitochondrial apoptosis pathways are the main types of apoptosis activated by drugs to eradicate harmful effects of disease [10]. Aloperine is capable of activating both types of apoptosis in multiple diseases. Aloperine mediated apoptosis suppressed the growth of various cancer cells, including osteosarcoma, colon cancer, multiple myeloma, pancreatic cancer, breast cancer, liver cancer, hepatoma, and glioma [11,12,13,14]. Interestingly, aloperine exhibited anti-apoptotic activity to improve disease conditions. Aloperine attenuated apoptosis to attain curative effects in ischemia and reperfusion (IR) induced renal injury, H_2_O_2_ induced injuries to neuronal cells, nucleus pulposus cells, ARPE-19 cells, and cerebral IR injury mice models (Table 1) [15,16,17,18,19].

Aloperine could stop cell cycle progress to inhibit the growth of tumor cells. The cell cycle is a series of events vital for cell division and the generation of two daughter cells. It mainly has four phases, including G1, S, G2, and M. Cell cycle is targeted by different chemo-preventive drugs to control cancer [20]. Literature review showed that aloperine arrested the cell cycle at different phases to inhibit the growth of multiple tumors, including prostate cancer, lung cancer, thyroid cancer, hepatocellular carcinoma, and colon cancer [14,21,22,23,24]. Moreover, aloperine can produce anti-invasion and anti-migration effects in different cancers by targeting the protein components of migration and invasion-promoting signaling mechanisms (Table 1) [25,26].

Autophagy, a degradative process, is responsible for removing abnormal or unnecessary components of cells. Aloperine could also modulate autophagy to improve pathological conditions like leukemia and thyroid cancer (Table 1) [7,27].

Cellular signaling mechanisms are a series of chemical processes which govern cell growth and survival. A single molecule or a group of molecules (signals) triggers the activation of these chemical reactions. As needed, signaling molecules (hormones and growth factors) are generated in the body, and these attach to a specific receptor on the cell surface to initiate a corresponding signaling cascade to accomplish required functions [28]. Aberrations in signaling mechanisms due to internal or external factors could develop multiple diseases. Many therapeutic strategies target molecules of potentially dysregulated signaling mechanisms to prevent or control disease progression. A literature review showed that aloperine is also one of the potent modulators of signaling mechanisms. Aloperine has been reported to inhibit the PI3K/Akt/mTOR signaling to attenuate the adverse effects of diseases like acute kidney disease, inflammatory diseases, and different types of cancer (Table 2) [11,14,15,29,30].

Moreover, aloperine altered the levels of components of NF-κB [18,31], Nrf2 [19,32], and Ras [33] signaling pathways to produce remedial effects against several diseases (Table 2). In this review, we summarize the current knowledge on the modulatory effects of aloperine on critical biological processes and signaling mechanisms. This study may provide helpful insight into understanding the management of disease-causing aberrations in signaling mechanisms, and it may aid in the development of new molecular mechanisms targeting treatment options in the future.

## 2. Regulation of Apoptosis

Apoptosis is one of the significant types of cell death [34], mainly directed by caspases (cysteine proteases). Apoptosis occurs by two main pathways: the extrinsic and intrinsic pathways.

Apoptosis is complex, energy-dependent process, and it is crucial in removing dying or unwanted cells in normal conditions. Apoptosis is one of many therapeutic agents’ common mechanisms of action [10,35].

The extrinsic apoptosis or death receptors pathway works by binding death receptors with specific ligands. This binding enables the recruitment of Fas-associated death domain (FADD), which could bind to Fas, TRAIL-R1/2, or TNFR1. This interaction causes the activation of downstream events, which ultimately leads to the activation of caspase 8. Activated caspase 8 brings about apoptosis either by directly activating caspases cascade (Type I) or indirectly by cytochrome c mediated activation of a caspase cascade (Type II) [36].

The intrinsic apoptotic pathway or mitochondrial apoptotic pathway is activated in response to context-dependent stimuli. It causes the release of cytochrome c to the cytosol. Cytochrome c undergoes ATP-dependent binding with protease activating factor-1 (Apaf-1), which results in apoptosome formation. The apoptosome activates Caspase-9, which activates caspases 3,6,7 to carry out apoptosis [12].

Aloperine proved to be a potent inducer of apoptosis. One study reported that aloperine treatment caused apoptosis in U266 and MM.1S myeloma cells by activating the extrinsic apoptosis pathway. Activation of caspases 8/9/3 through aloperine therapy executed apoptosis. In this study, aloperine was found to activate the caspase by inhibiting the anti-apoptotic cFLIP [22]. The apoptotic role of aloperine is also investigated in prostate cancer cells, which showed that aloperine induced apoptosis by changing the Bax/Bcl-2 ratio. It causes an increase in Bax (pro-apoptotic) and a decrease in Bcl-2 (anti-apoptotic). The change in the concentration of these apoptosis-related proteins activated caspase 3, which ultimately induced apoptosis in PC3, DU45, and LNCaP prostate cancer cells. These findings indicate that aloperine brought about apoptosis through the extrinsic apoptosis pathway [23]. Aloperine executed apoptosis in hepatocellular carcinoma cells. Aloperine treatment augmented cytochrome c level in the cytoplasm of hepatocellular carcinoma cells.

Moreover, it caused the cleavage of caspase-9, caspase-3, and PARP and raised the levels of cleaved-caspase-9, cleaved-caspase-3, and cleaved-PARP (poly ADP ribose polymerase). This series of events lead to the apoptosis of liver cancer cells. The outcomes of this study indicate that aloperine promoted apoptosis in HCC cells through the intrinsic apoptotic pathway [11].

The apoptosis induction effects of aloperine in osteosarcoma, colon cancer, breast cancer, glioma, and leukemia cells were determined. In these studies, the outcomes of western blotting and PCR experiments showed that aloperine treatment caused an increase and decrease in the levels of Bax and Bcl-2, respectively, and it also elevated cleaved caspase 3 level [7,11,14,26,37]. Similarly, aloperine inhibited Bcl-2 activity in bladder and NSCLC cells and caused apoptosis [24,33]. Since Bcl-2 protein and cleaved caspase-3 are the main components of the intrinsic apoptotic pathway [38,39], modulations in their levels showed that aloperine brought about apoptosis in OS cells through the intrinsic apoptotic pathway.

Aloperine also triggered apoptosis in human thyroid carcinoma. IHH-4 and KMH-2 cells were found more susceptible to aloperine-induced programmed cell death. Aloperine treatment activated caspase-3 and PARP in a dose- and time-dependent manner. It also increased the levels of cleaved caspase-9 in IHH-4 and KMH-2 cells. Additionally, aloperine-treatment activated caspase-8 in KMH-2 cells. These outcomes indicate that aloperine activated intrinsic and extrinsic apoptosis pathways in human thyroid carcinoma cells [30].

The circNSUN2 RNA could promote cancer progression by binding to various RNA binding proteins. Regulation of the formation of circNSUN2 RNA-Protein complex could prevent cancer progression. Aloperine could inhibit the activity of circNSUN2 and counteract the tumor-promoting effects of circNSUN2. These findings suggest that aloperine treatment attenuated cell proliferation and increased the apoptosis in colorectal cancer cells via regulating the circNSUN2/miR-296-5p/STAT3 pathway [40].

Acute kidney disease resulting from renal ischemia and reperfusion (IR) damage is associated with high morbidity and mortality [41]. Tubular cell death frequently occurs in acute renal injury caused by IR [42]. The IR insult could raise caspase-3 levels and induce apoptosis in tubular cells. Interestingly, Hu et al. reported that aloperine treatment reduced tubular cells apoptosis in IR mice models. Protein expression analysis revealed a 1.3-fold reduction in caspase 3 levels in aloperine treated IR mice models compared to untreated mice models. These findings indicate that the treatment of aloperine could reduce apoptosis in tubular cells in IR mice [15].

This conclusion contradicts research in tumor cells where aloperine mainly promotes apoptosis in cancer cells. This variation in the outcome of aloperine treatment might be due to the differing aloperine doses utilized in cancer therapy.

Hydrogen peroxide (H_2_O_2_) exposure can trigger apoptosis in N2a/Swe.D9 neuronal cells by activating the mitochondrial apoptotic pathway. Zhao et al. reported that aloperine inhibited the H_2_O_2_ mediated apoptosis in N2a/Swe.D9 cells. Hydrogen peroxide treatment promoted the release of cytochrome C from mitochondria to cytosol. Additionally, it decreased the Bcl-2 levels and activated caspase 3, but aloperine treatment reversed this apoptosis triggering effects and prevented N2a/Swe.D9 cells death [43]. Moreover, Ren et al. reported the inhibition of H_2_O_2_-mediated apoptosis in nucleus pulposus cells by aloperine. Hydrogen peroxide exposure induced apoptosis by increasing the caspase-9 activity in nucleus pulposus cells, but aloperine treatment inhibited the apoptosis of nucleus pulposus cells by attenuating the activity of caspase-9 [44].

**Figure 1:**
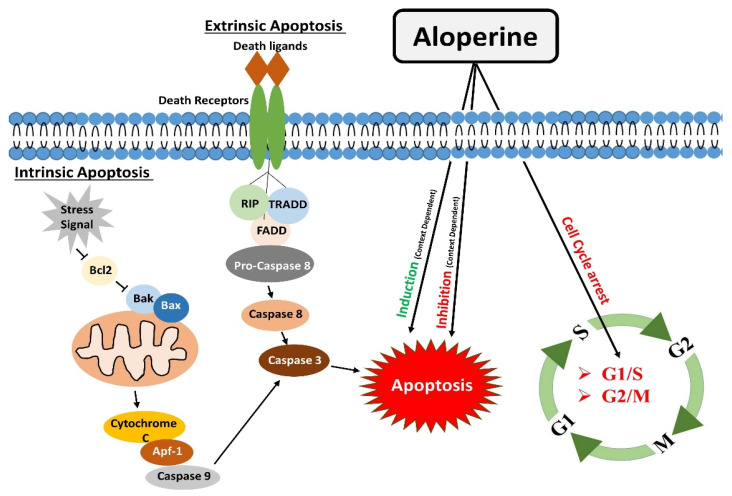
Modulatory effects of aloperine on apoptosis and cell cycle.

Similarly, Zhang et al. also reported the anti-apoptotic effects of aloperine in H_2_O_2_ treated ARPE-19 cells. Hydrogen peroxide facilitated a decrease in Bcl-2 levels, and increased caspase 3 activity was mitigated by aloperine [19]. Furthermore, Li et al. evaluated the effects of aloperine in middle cerebral artery occlusion (MCAO)/reperfusion injury rat models. Brain sections of Rats models with cerebral IR injury showed a significant population of apoptotic cells and decreased Bcl-2 protein levels. Interestingly, aloperine treatment inhibited the apoptosis effects in rat models under investigation [16]. This finding shows that aloperine could regulate apoptotic pathways in a context and disease-dependent manner (Figure 1).

## 3. Modulatory Effects on the Cell Cycle

During the cell growth and division, it undergoes a series of events known as the “cell cycle”. G1, S, G2, and M are the four main cell cycle phases. In the G1 phase, the cellular machinery makes preparation to divide. In cell division, the cell enters the S phase, during which it duplicates all of its genetic material. Hence, the suffix “S” stands for DNA synthesis. During the G2 stage, the arrangement and packaging of already duplicated genetic material are completed. The cell cycle moves to the next phase of the cell cycle. M phase is the next step in which cells physically divide into two daughter cells, and the copies of genetic material are distributed to newly formed daughter cells. At the end of the M phase, the cell cycle completes [45]. Specific serine/threonine-protein kinase regulates each cell cycle phase, known as cyclin-dependent protein kinases (CDKs). Cell cycle phase-specific CDKs make complexes with cyclin regulatory subunits and facilitate the cell cycle progression from one phase to the next [46]. Many drugs achieve their therapeutic effects by targeting the cell cycle. Blocking the cell cycle at different phases results in cell growth inhibition.

A review of the literature exhibited that aloperine can effectively block the transition of the cell cycle at different stages. Cell cycle analysis of aloperine treated prostate cancer (PC) cells showed a high proportion of cells at the G1 phase. Further, western blotting analysis revealed increased p53 and p21 proteins, which confirmed that aloperine caused G1 phase cell cycle arrest in PC cells [22]. Previously, our research group conducted a study in NSCLC cells. We also found that aloperine could cause G1 phase cell cycle arrest in NSCLC cells. Our study showed that aloperine treatment upregulated the p53 and p21 proteins and downregulated the levels of Cyclin E, CDK2, pRb, and E2F1 proteins. By modifying the levels of G1 phase controlling proteins, aloperine achieved G1 phase cell cycle arrest in NSCLC cells [24].

Liu et al. reported that aloperine stopped the G2/M phase transition of the hepatocellular carcinoma cell cycle. Flow cytometry analysis of aloperine treated cells showed a high number of cells at the G2/M phase. Expression analysis exhibited low cdc25C, cdc2, and cyclin B1 proteins in aloperine treated Hep3B and Huh7 cells [23]. Moreover, G2/M phase arrest has also been observed in aloperine treated human colon cancer HCT116 cells. Cell cycle histograms showed elevated peaks at the G2/M phase of the cells cycle. The expression pattern of G2/M phase associated proteins p53, p21, cyclin D1, and B1 confirmed G2/M phase cells cycle arrest in HCT116 cells [14].

Furthermore, a study reported that aloperine executed G2/M phase cell cycle arrest in SNU-182 cancer cells. Propidium Iodide (PI) staining showed a high population of cells at the G2/M phase of the cell cycle. Interestingly, this study reported that overexpression of GRO1 oncogene reversed the cell cycle arresting effects of aloperine in SU-182 liver cancer cells. This finding indicates that aloperine may cause cell cycle arrest in SU-182 cells via downregulating GRO1 oncogene [21]. However, further investigations are needed to affirm this inference.

On the contrary, aloperine treatment could not cause cell cycle arrest in IHH-4, 8505c, and KMH-2 thyroid cancer cells. There were no apparent changes in cell cycle histogram patterns [30]. This finding is inconsistent with the findings of studies conducted in other cell types, and this inconsistency might be due to differences in the genetic makeup of different cell types (Figure 1).

## 4. Modulation of Autophagy

Autophagy is an evolutionarily conserved catabolic process that operates to degrade/remove undesirable cellular components, such as truncated or long-lasting proteins and unnecessary organelles [47,48]. Macro-autophagy, micro-autophagy, and chaperone-mediated autophagy are the three kinds of autophagy that have been described so far. Among all types, macro-autophagy is perhaps the most well investigated. The first step in autophagy is the formation of phagophores, which encloses truncated proteins/defective organelles. Phagophores undergo elongation and form a double membranous vesicle known as an autophagosome. These double membranous vesicles move towards and fuse with lysosomes to form autolysosomes. Finally, by the action of lysosomal enzymes, unwanted material is degraded, and recycled products are used to form new structures or used as energy sources [49].

Autophagy is a vital degradation process that maintains cellular homeostasis [50,51]. Many drugs, synthetic or natural, target autophagy to exert their therapeutic effects.

Lin et al. conducted a study in HL-60 leukemia cells and evaluated the effects of aloperine treatment on autophagy. They showed that aloperine treatment for 18 h triggered the development of autophagic vacuoles. Acridine orange staining showed that the formation of autophagic vacuoles improved with the increase in the aloperine dosage. These findings demonstrated that aloperine could promote autophagy in HL-60 cells [7]. Moreover, aloperine exerted modulatory effects on autophagy were evaluated in thyroid cancer cells. Three types of thyroid cancer cells, KMH-2, IHH-4, and 8505c cells, were employed in this study.

**Figure 2:**
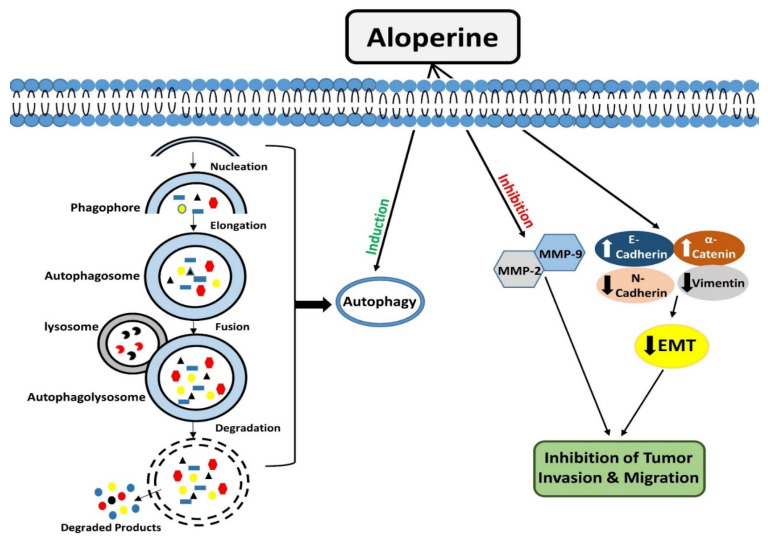
Modulatory effects of aloperine on autophagy and tumor cell invasion & migration.

Interestingly, it was observed that aloperine treatment enhanced autophagosome formation and autophagic activity in KMH-2 and IHH-4 cells, but it did not produce such outcomes in 8505c cells. The expression analysis of LC3-II and p62 markers showed that aloperine blocked autophagic flux in 8505c cells [27]. The underlying molecular mechanism for aloperine to exhibit this dual role needs further elucidation (Figure 2).

## 5. Inhibitory Effects of Aloperine on Tumor Cell Invasion and Migration

Tumor cells can invade their surrounding or distant tissues. Metastasis is a multistep process in which tumor cells escape from their original site, enter the blood circulation, and travel to distant organs of the body [52]. Matrix metalloproteinases (MMPs), zinc-dependent endopeptidases, facilitate tumor cell invasion by remodeling and degrading the extracellular matrix [53,54,55]. Circulating epithelial cancer cells could undergo epithelial-mesenchymal transition (EMT) to achieve invasion and metastasis. During this process, the innate differentiation properties of epithelial cells are lost, and these acquire phenotype like mesenchymal cells, which help them migrate and invade surrounding tissues [56,57].

Tiani et al. determined the effects of aloperine on the migration and invasion of breast cancer cells. The outcomes of wound healing and trans-well assays showed that aloperine halted the motility and migration of MCF-7 and MDA-MB-231 cells in a dose-dependent manner. Further, the levels of metalloproteinases were also evaluated, which showed that aloperine downregulated MMP2 and MMP9. Collectively these findings showed that aloperine has anti-migratory and anti-invasive effects in breast cancer cells [26].

Another study reported that aloperine could inhibit migration and invasion of liver cancer cells. They showed that aloperine treatment coupled with GROa knockdown in SNU-18 cells significantly reduced migration and cell invasion by 70% compared to untreated cells. Additionally, aloperine upregulated the mRNA expression of EMT inhibitory molecules, like E-cadherin and α-catenin, while decreasing the mRNA levels of EMT-promoting molecules like fibronectin and vimentin [21].

Excessive proliferation of tumor cells could create a hypoxic condition in the tumor microenvironment [58]. Hypoxia could promote invasiveness and migration of many types of tumor cells, including bladder cancer cells. T24 bladder cancer cells under hypoxic conditions were treated with aloperine to elucidate its invasion and migration inhibitory function. Trans-well assay exhibited that hypoxia significantly raised the migration rate of tumor cells, but aloperine inhibited migration. Similarly, aloperine also inhibited hypoxia-induced EMT by increasing the levels of E-cadherin and attenuating the levels of N-cadherin and vimentin. Additionally, the aloperine mediated downregulation of EMT promoting transcription factors (snail and twist1) further strengthened its EMT reversal role in bladder cancer cells [25] (Figure 2).

**Table 1:** Aloperine mediated modulations in biological mechanisms.

Apoptosis						
Pathological Conditions	Cell Lines	Animal Model	Dosage		Regulatory Effects of Aloperine	Ref.
			In Vitro (µM)	In Vivo		
Multiple Myeloma	U266 and MM.1S	SCID NOD mice	50/100/250/500	20 mg/kg	Induced Caspase-dependent apoptosis	[12]
Prostate cancer	PC3, DU145 and LNCaP	BALB/C mice	100/200	30 mg/kg	Induced Caspase dependent apoptosis	[22]
Hepatocellular carcinoma	Hep3B and Huh7	Zebrafish embryo	200/350/500	100 µM, 150 µM	Induced Mitochondria-dependent apoptosis	[23]
Osteosarcoma	MG-63 and U2OS	---------	100/200	---------	Induced Mitochondria-dependent apoptosis	[11]
Colon cancer	HCT116	---------	250/500	--------	Induced Mitochondria-dependent apoptosis	[14]
Breast cancer	MCF-7 and MDA-MB-231	---------	100/200/400	---------	Induced Mitochondria-dependent apoptosis	[26]
I/R-Induced Renal Injury	RAW264.7 and HK2	C57BL/6 mice	500	50 mg/kg	Inhibition of Apoptosis	[15]
Thyroid Cancer	IHH-4,8505c and KMH-2	---------	100/200	---------	Induced Caspase-dependent apoptosis	[30]
Leukemia	HL-60	---------	50/100	---------	Induced Mitochondria-dependent apoptosis	[7]
Alzheimer’s disease	N2a/Swe.D9	---------	100	---------	Induced Mitochondria-dependent apoptosis	[43]
Non-small cell lung cancer	H1944 and NCI-H1869	BALB/C nude mice	250	30 mg/kg	Induced Mitochondria-dependent apoptosis	[24]
Intervertebral disc degeneration	Nucleus Pulposus cells	Sprague-Dawley rats	100	---------	Inhibition of Apoptosis	[44]
Bladder Cancer	EJ cells	---------	25/50/100	---------	Induced Mitochondria-dependent apoptosis	[59]
OGD/RP neuronal injury	Hippocampal Neuronal cells	Sprague-Dawley rats	100/200/400	---------	Inhibition of Apoptosis	[60]
Colorectal Cancer	SW480 and HT29	---------	200/400/800/1000	---------	Induced Mitochondria-dependent apoptosis	[40]
Early brain injury	---------	Sprague-Dawley rats	---------	75/150 mg/kg	Inhibition of Apoptosis	[17]
I/R-Induced Cerebral injury	---------	Sprague-Dawley rats	---------	2/25/50 mg/kg	Inhibition of Apoptosis	[16]
Retinal pigment epithelial cells injury	ARPE-19	---------	6.25/12.5/25	---------	Inhibition of Apoptosis	[19]
DSS-Induced Colitis	Jurkat Cells	C57BL/6 mice	250/500	40 mg/kg	Inhibition of Apoptosis	[29]
Microembolisation-Induced cardiac Injury	---------	Sprague-Dawley rats	---------	200 mg/kg	Inhibition of Apoptosis	[61]
**Cell Cycle**						
Prostate cancer	PC3, DU145 and LNCaP	BALB/C mice	100/200	30 mg/kg	G1 phase arrest	[22]
Hepatocellular carcinoma	Hep3B and Huh7	Zebrafish embryo	200/350/500	100 µM, 150 µM	G2 phase arrest	[23]
Colon cancer	HCT116	---------	250/500	---------	G2 phase arrest	[14]
Thyroid Cancer	IHH-4,8505c and KMH-2	---------	100/200	---------	No impact on Cell Cycle	[30]
Non-small cell lung cancer	H1944 and NCI-H1869	BALB/C nude mice	250	30 mg/kg	G1 phase arrest	[24]
Liver cancer	SNU-182	---------	5	---------	G2 phase arrest	[21]
**Autophagy**						
Thyroid Cancer	KMH-2 and IHH-4	---------	200	---------	Autophagy induction	[27]
Thyroid Cancer	8505c	---------	200	---------	Autophagy inhibition	[27]
Leukaemia	HL-60	---------	50/100	---------	Autophagy induction	[7]
**Migration and Invasion**						
Breast cancer	MCF-7 and MDA-MB-231	---------	100/200/400	---------	Inhibition of Migration and Invasion	[26]
Liver cancer	SNU-182	---------	5	---------	Inhibition of Migration and Invasion	[21]

## 6. Modulatory Effects on PI3K/Akt/mTOR Signaling

Phosphoinositide 3-kinases (PI3K) belongs to the lipid kinase family. These mainly perform the phosphorylation of phosphatidylinositol (PtdIns) lipids at 3 hydroxyl group of their inositol ring [62]. Various external stimuli, such as growth factors, cytokines, and hormones, drive their activation. Phosphoinositide 3-kinases activation occur through a series of events. It starts when external stimuli like epidermal growth factor (EGF), platelet-derived growth factor, and insulin-like growth factor [63,64] bind to the N-terminal extracellular domain of plasma membrane-spanning receptor tyrosine kinase (RTK). It leads to the phosphorylation of tyrosine residues of RTK towards the cytoplasmic region, linker molecule, and p85SH2 subunit of PI3K. The binding of a phosphorylated tyrosine residue in RTK and domain results in the recruitment of PI3K to RTKs and directs the allosteric activation of PI3K. Besides RTKs, G-protein coupled receptors can also activate PI3K. The activated PI3K could activate various downstream proteins to perform multiple functions. The PI3K/Akt signaling pathway is one of the main pathways that regulate cellular physiological functions, including cell survival, proliferation, and invasion [65,66,67,68].

The modulations in PI3K/Akt signaling pathway activity are associated with different kinds of diseases. Various drugs, especially anti-cancer drugs, target PI3K/Akt signaling pathways to combat diseases [69].

Activation of the PI3K/Akt signaling pathway has been linked to poor prognosis, enhanced progression, and development of severe pathological complications in HCC patients [70,71]. Thus, targeting the PI3K/Akt signaling pathway could help treat HCC [72]. Liu et al. investigated the effects of aloperine treatment on PI3K/Akt in HCC cells. Aloperine treatment lowered the expression of p110, p85, and Akt. It is known that phosphorylation at Thr308 and Ser473 drives Akt activation. Aloperine inactivated Akt by preventing Ser 473 phosphorylation and reducing p-Akt (Ser473) levels but did not change p-Akt (Thr308) levels. The findings of this study show that aloperine inhibited the PI3K/Akt pathway by attenuating the levels of its components (p110, p85, p-Akt (Ser473)). Aloperine mediated inhibition of PI3K/Akt pathway resulted in suppression of HCC [23].

One of the most commonly occurring bone tumors in children and adults is osteosarcoma, which develops due to improper differentiation of MSC [73,74]. Chen et al. investigated the effects of aloperine on the PI3K/Akt signaling pathway in osteosarcoma cells. Western blotting and qRT-PCR were performed on aloperine treated OS cells which revealed an enormous decrease in the expression levels of both PI3K and p-Akt1. The outcomes of this study showed that aloperine killed the OS cells by suppressing the PI3K/Akt pathway [11].

Colon cancer is among the top cancers with a high mortality rate [75]. Zhang et al. treated HCT116 colon cancer cells with aloperine and reported inhibition of HCT116 cells growth by aloperine. Furthermore, to elucidate the underlying molecular mechanism, treated cells were analyzed for changes in the expression of components of PI3K/Akt and JAK/Stat3 signaling pathways. Both these pathways play a vital role in tumor cell survival. Expression analysis revealed that aloperine treatment reduced Stat3 and PI3KC3 levels in a dose-dependent manner. Thus, aloperine treatment inhibited HCT116 cells growth by blocking PI3K/Akt and JAK/Stat3 pathways [14].

Acute kidney disease resulting from ischemia and reperfusion (IR) injury is associated with a high degree of morbidity and mortality [76]. Ischemia and reperfusion injury triggers activation of pro-inflammatory cytokines, which produces an inflammatory response at the injury site [77,78,79,80]. Hu et al. reported that aloperine could prevent IR mediated acute renal injury in mice models. Investigation of the molecular mechanism responsible for aloperine mediated protection. It was noted that IR injury activated PI3K/Akt/mTOR pathway, but aloperine treatment significantly reduced the levels for phosphorylated PI3Kp85, Akt, and mTOR. Thus, aloperine treatment inhibited the activities of PI3K and its downstream targets Akt and mTOR to protect IR mediated acute renal injury in mice models [15].

Thyroid cancer is considered one of the primary cancers of endocrine glands [81]. PI3K/Akt pathway could facilitate the occurrence of carcinomas of thyroid origin [82]. Yu et al. conducted a study in IHH-4 and KMH-2 thyroid cancer cells to evaluate the effects of aloperine on Akt activity in these cells. Expression analysis showed that aloperine treatment significantly downregulated the p-Akt and caused the death of thyroid cancer cells by suppressing the Akt pathway [27].

Colitis is the most frequent type of inflammatory bowel disease [83]. Mucosal immune dysfunction could trigger modulations in T cells’ activities, which play a vital role in the pathogenesis of inflammatory bowel diseases [84]. PI3K/Akt/mTOR signaling pathway is known to positively and negatively regulate pro-inflammatory T helper cell 17 (Th17) and anti-inflammatory regulatory T-cells (Tregs), respectively [85,86]. Fu et al. treated colitis mice models with aloperine and observed that aloperine treatment attenuated inflammation and improved colitis in mice models. Investigation of the molecular mechanism responsible for colitis improvement revealed that aloperine treatment in Jurkat cells attenuated the levels of p-PI3K p85, p-Akt, and p-mTOR (major molecules of PI3K/Akt/mTOR pathway). These findings indicate that aloperine alleviated colitis by suppressing PI3K/Akt/mTOR pathway in colitis mice models and Jurkat cells [29].

It is well known that both Akt and ERK function by promoting cell survival, proliferation, and metabolism, and their activities could facilitate tumor progression [87,88]. Ling et al. employed aloperine to evaluate its anti-tumor effects in prostate cancer. Aloperine effectively inhibited PC growth. Treated prostate cancer cells showed reduced levels of p-Akt and p-ERK. In this study, aloperine achieved its tumor-suppressive effects under Akt and ERK signaling inhibition [22].

On the contrary, Mao et al. reported that aloperine could activate PI3K/Akt pathway to alleviate myocardial injury in rats. Coronary micro-embolization (CME) is a common complication of acute coronary syndrome [89]. Coronary micro-embolization could cause the death of cardio-myocytes, and it can also lead to systolic dysfunction [90]. Mao et al. utilized Aloperine to treat CME-mediated myocardial injury in rats. Aloperine alleviated myocardial injuries like myocardial microinfarction and cardiomyocytes necrosis, and improved cardiac function in rats. Aloperine treatment increased p-Akt levels, activating the PI3K/Akt pathway, preventing myocardial necrosis, and protecting against myocardial injury [61].

**Figure 3:**
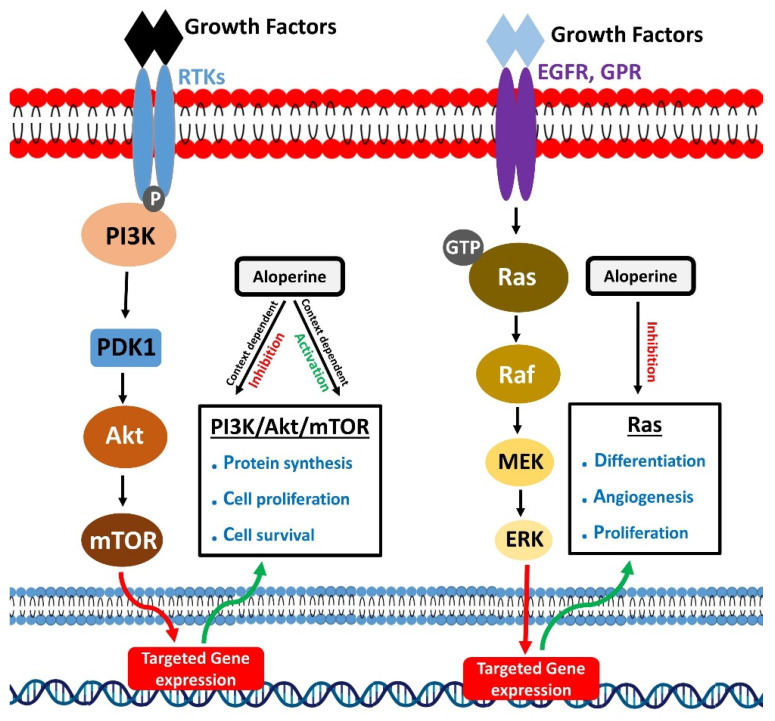
Modulatory effects of aloperine on PI3K/Akt/mTOR and Ras signaling.

Similarly, PI3K/Akt activation by aloperine could prevent cerebral ischemia in mice models. Cerebral ischemia/reperfusion (IR) injury is fatal for brain functioning, and it is also considered a frequent cause of stroke [91,92]. Cerebral IR injury could promote neuronal injury by producing conditions like inflammation and oxidative stress [93,94]. Li et al. reported that aloperine produced neuroprotective effects and improved vertebral injury in cerebral IR injury rat models. A study of molecular mechanism revealed that aloperine treatment reversed the cerebral IR injury mediated inhibition of the PI3K/Akt pathway. Aloperine increased p-PI3K, p-Akt levels which inhibited neuronal cell death and improved cerebral function in rats (Figure 3) [16].

## 7. Inhibition of NF-κB Signaling

Transcription of several immune and inflammatory reactions related genes is controlled by the nuclear factor-kB (NF-kB) [95]. RelA (p65), RelB (p65), c-Rel, NF-kB1 (p50), and NF-kB2 (p52) are structurally related members of this family. All five members bind to a specific DNA region, known as kB enhancers, to control transcription of target genes [96]. IkB proteins are ankyrin repeat-containing inhibitory proteins, which sequester NF-kB proteins in the cytoplasm of cells [97]. IkBα is currently one of the most studied and important members of the IkB proteins family. Two signaling pathways, canonical and non-canonical, mainly activate NF-κB. Canonical and Non-canonical pathways control inflammatory and immune responses through different signaling mechanisms [98,99]. Different stimuli, including ligands of Pattern recognition receptors (PRRs), TNF receptors (TNFR) superfamily members, T and B-cell receptor and cytokines receptors, could activate the canonical NF-κB pathway through a series of events [100]. These stimuli activate IκB kinase (IKK), which phosphorylates and degrades IkBα.

Consequently, nuclear translocation of members of canonical NF-κB pathway occurs quickly, where they perform their transcription regulatory function [101,102]. On the other hand, the non-canonical NF-κB pathway activates in response to particular stimuli, including LTβR, BAFFR, CD40, and RANK, which are the ligands of the members of the TNFR superfamily. An NF-κB-inducing kinase (NIK) plays a leading role in activating the non-canonical NF-κB pathway. NF-κB-inducing kinase, in combination with IKK, causes phosphorylation of p100. This processing of p100 produces p52, making p52/RelB complex, translocating to the nucleus to perform its functions [103,104]. Nuclear factor-kB is known to control immune and inflammatory responses. It also plays a prominent role in differentiating inflammatory T cells and activating inflammasomes [105,106,107]. Dysregulated NF-κB signaling has been associated with the onset of multiple inflammatory diseases.

Lipopolysaccharides (LPS) are mainly present in the cell wall of gram-negative bacteria, and LPS tend to cause the release of pro-inflammatory cytokines and trigger inflammatory response [108,109]. Inflammation is a primordial body’s response to stress conditions, but excessive inflammation could produce harmful effects like tissue injury, systemic failure, respiratory failure, or even death [110]. Ye et al. introduced LPS to macrophages to induce inflammatory responses in macrophages and treated these activated macrophages with aloperine to assess the anti-inflammatory effects of aloperine treatment. Aloperine lessened the inflammatory responses in LPS-activated macrophages by suppressing the release of TNF-α, IL-6, and Interleukin-17A pro-inflammatory cytokines. Further analysis showed that aloperine executed anti-inflammatory effects by inhibiting the NF-κB pathway. Aloperine treatment inhibited degradation of IkB and prevented the nuclear translocation of p65, hence inhibiting NF-κB pathway [111].

Chronic inflammation of air passages could lead to bronchial asthma [112]. An increase in IL-4, IL-5, and IL-13, and decrease in the levels of interferon-γ facilitate eosinophils entry and release of immunoglobulin E (IgE) into the lungs [113]. Asthma could produce complications like eosinophils mediated inflammation in the air passage, mucus hypersecretion, and airway hyper-responsiveness (AHR) [112]. Wang et al. employed aloperine to investigate its protective effects against asthma. Aloperine treatment improved asthma conditions in mice models by lowering inflammatory cells infiltration and reducing IL-4, IL-5, and IL-13 and IgE levels. Moreover, aloperine blocked the cytoplasm to nucleus translocation of NF-κB related proteins and ensured the activity of IκBα. This study showed that aloperine mediated inhibition of the NF-κB pathway was one of the significant reasons for alleviating asthma in mice models [18].

Neuropathic pain is a chronic and frequent condition originating from lesions or diseases of the somatosensory nervous system [114]. Chronic constriction injury (CCI) mediated neuropathic pain mice models were given aloperine treatment, which produced antinociceptive effects in mice models. NF-κB and its related inflammatory mediators could promote neuropathic pain [115]. In line with this evidence, Xu et al. observed elevation in levels of the NF-κB and its downstream inflammatory mediators following induction of CCI mediated neuropathic pain in mice models, while aloperine treatment reversed this increment. Outcomes of this study indicate that inhibition of NF-κB signaling is one of the critical events in the course of neuropathic pain alleviation [31].

Another study reported the inhibition of the NF-κB pathway by aloperine in nucleus pulposus cells. Ren et al. in their study, evaluated the protective effects of aloperine against oxidative stress-mediated injury in nucleus pulposus cells (NPC). In the body, injury or improper functioning of NPCs could lead to intervertebral disc degeneration [116]. Nucleus pulposus cells extracted from mice models were subjected to H_2_O_2_ treatment, which produced inflammation. Expression analysis exhibited that H_2_O_2_ treatment-induced inflammation by upregulating the NF-κB pathway. Aloperine treatment imparted anti-inflammatory effects and enhanced NPCs viability by inhibiting NF-κB pathway [31].

Postmenopausal osteoporosis could cause bone fractures and is considered one of the leading causes of disability and mortality in older women [117]. Bone resorption by osteoclasts is mainly attributed to osteoporosis. Osteoclasts are mainly derived from monocyte/macrophage differentiation. Nuclear factor κB ligand-receptor activator (RANKL), a cytokine, plays a vital role in the course of cellular differentiation [118,119,120]. Hu et al. conducted differentiation of BMM (bone marrow derived macrophages) to osteoclast and added aloperine during the passage of differentiation. Results showed that aloperine negatively impacted osteoclast differentiation by inhibiting the osteoclastogenesis-promoting genes. During osteoclast formation, RANKL activates various signaling pathways, including NF-κB. Western blotting analysis showed that aloperine addition inhibited the NF-κB pathway and suppressed osteoclast formation [121].

**Figure 4:**
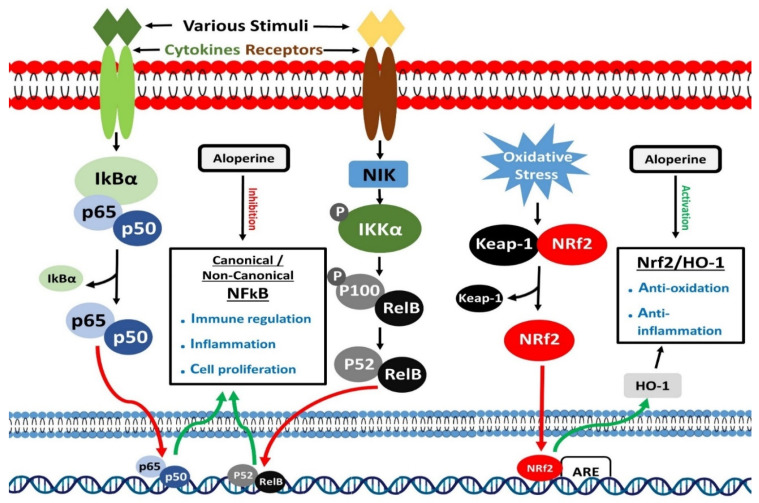
Modulatory effects of aloperine on NF-κB and Nrf2 signaling.

Pulmonary arterial hypertension (PAH) is characterized by high arterial blood pressure [122]. Several factors contribute to its occurrence, but inflammatory cells mediate imbalance between vasodilator and contractile factors is considered the leading cause of PAH [123]. Li et al. utilized aloperine to treat PAH mice models and reported that aloperine lessened PAH severity in mice models by improving hemodynamic parameters, protecting vascular endothelial cells, reducing ventricular hypertrophy, and inhibiting inflammatory responses. Western blotting results showed that aloperine targeted inflammation-inducing signaling pathways, mainly NF-κB in mice models, to curb PAH-associated adverse effects (Figure 4) [124].

## 8. Activation of Nrf2 Signaling

Cap ‘n’ collar (CNC) transcription factors are the members of the basic region leucine zipper (bZip) transcription factors family. The nuclear factor erythroid 2 (NFE2)-related factor 2 (Nrf2) is one of the essential members of CNC transcription factors [125]. To cope with oxidants and electrophiles, Nrf2 promotes activation of various drug-metabolizing enzymes, including glutathione S-transferase (GST) and NAD(P)H: quinone oxidoreductase 1 (NQO1) [126,127]. A DNA region known as the antioxidant response element (ARE), which resembles the NFE2-binding motif, is required for Nrf2 mediated activation of drug-metabolizing enzymes [128]. Activated enzymes play an essential role in detoxifying and removing chemical substances from either internal or external sources. In response to oxidants and electrophiles, Nrf2 acts as a xenobiotic-activated receptor (XAR) and protects the body from chemical toxicities [129]. In recent investigations, the Nrf2 mediated protection from oxidant stress has emerged as a prominent function of Nrf2 [130]. In many studies, therapeutic agent led elevation of Nrf2 activity contributed to protecting mice models from oxidative injury [131]. Many Nrf2 targeted ARE-containing genes have been recognized through genome-wide search. These genes mainly maintain oxidant homeostasis and drug metabolism [132]. Heme oxygenase-1 (HO-1) gene is one of the critical Nrf2 target genes [133]. It encodes an enzyme that catalyzes the conversion of biliverdin to bilirubin and heme to carbon monoxide (CO) and free iron [134]. Many studies have extensively investigated the upregulation of the HO-1 gene by Nrf2 and the anti-oxidative function of the Nrf2/HO-1 axis. Moreover, Nrf2 is known to activate >600 genes, and the proteins encoded by the majority of these genes perform cyto-protective functions and prevent the body from cancer, inflammatory and neurodegenerative diseases [135,136,137].

Age-related macular degeneration (AMD) is one of the leading causes of vision loss in elderly patients [138,139]. Among many factors, oxidative stress is an important inducer of AMD [140,141]. The retinal pigment epithelium (RPE) layer separates neuro-retina from choriocapillaris, supports photoreceptors, produces growth factors, and ensures immune privilege for retinal cells [142]. Oxidative stress-mediated impaired functioning of RPE is a frequent hallmark of age-related macular degeneration [143]. Zhang et al. induced oxidative stress in human RPE (ARPE-19) cells through H_2_O_2_ exposure and treated these cells with aloperine. Outcomes of subsequent experiments revealed that aloperine attenuated oxidative stress. Moreover, aloperine elevated levels of nuclear Nrf2 and HO-1 proteins. This study concluded that aloperine mediated activation of Nrf2/HO-1 pathway minimized H_2_O_2_ mediated oxidative stress and protected ARPE-19 cells from possible harmful effects [19].

Prolonged hyperglycemia could induce oxidative stress that could negatively impact the functioning of Schwann cells [144]. This damage could impair axonal regeneration and axon atrophy and interfere with the nerve conduction velocity [145]. Chen et al. measured the anti-oxidative effects of aloperine in oxidative stress suffering RSC96 Schwann cells. High glucose treatment raised reactive oxygen species (ROS) and Malondialdehyde (MDA) levels, and aloperine reversed these levels. Western blotting analysis exhibited high Nrf2 and HO-1 proteins following aloperine treatment, which indicates that aloperine handled high glucose-induced oxidative stress in Schwann cells through Nrf2/HO-1 pathway activation [32].

Exogenous toxins could cause liver injury and alter liver functions by inducing oxidative stress and other cellular responses [146]. Xiong et al. prepared liver injury mice models employing Carbon tetra chloride (CCl_4_) treatment. Histological and biochemical analysis of mice samples showed that CCl_4_ treatment caused liver cell damage and raised liver enzymes (AST, ALT, ALP). Aloperine treatment alleviated damage to hepatocytes and restored elevated liver enzymes levels. It was noted that induction of oxidative stress by CCl_4_ was one of the main reasons for liver injury in mice models. Aloperine treatment raised Nrf2 and HO-1 levels, which produced anti-oxidative effects [147]. This study determined that aloperine dealt with liver injury by restraining oxidative stress.

Chronic air passage inflammation like asthma may arise due to oxidative stress [112]. An antioxidant response could help improve oxidative stress-mediated allergic air passage inflammation [148]. Wang et al. developed asthmatic mice models and treated these mice models with aloperine. Immuno-histochemical staining of tissue sections of aloperine treated asthmatic mice models showed larger Nrf2 and HO-1 areas around airways than control mice models. Similarly, expression analysis revealed higher NRF2 and HO-1 proteins levels than control mice models [18]. These findings suggest that aloperine can relieve asthma by triggering Nrf2/HO-1 pathway (Figure 4).

## 9. Inhibition of Ras Signaling

Ras proteins mainly belong to low molecular weight GTP-binding proteins superfamily [149]. These could regulate critical signaling pathways to control cell survival and proliferation [150]. Ras proteins are activated following binding to GTP [151]. Epidermal growth factor receptor (EGFR) and G-protein-coupled receptors promote RAS-GTP binding and activation of Ras [152]. Activated Ras proteins interact with effector molecules and activate downstream signaling mechanisms. Ras/Raf/MEK/ERK cascade is a crucial Ras regulated signaling mechanism [153]. GTP-bound Ras actives Raf kinases, which activate mitogen-activated protein kinase kinases 1 and 2 (MEK1/2). Next, MEK1/2 mediate phosphorylation and activation of ERK1/2 (Extracellular signal-regulated kinases 1 and 2). ERK1/2 or mitogen-activated protein kinases (MAPK) further phosphorylate various transcription regulators and control gene expression [154]. Besides the cascade mentioned above, Ras proteins could regulate other important signaling mechanisms to execute their cell growth and survival-promoting role.

Ras is an oncogene, which is usually present in the human genome. It can transform normal human cells into tumor cells. Approximately 30% of all tumors have exhibited mutations in Ras gene [155,156]. Mutations that lead to overexpression of the Ras gene have been found to promote growth, angiogenesis, and inhibition of apoptosis in tumor cells [157]. Many therapeutic agents have been investigated for their Ras inhibitory role in various cancers. Tian et al. employed aloperine to treat breast cancer cells and found that aloperine treatment induced tumor inhibitory effects by targeting Ras protein. Treated MCF-7 and MDA-MB-231 breast cancer cells showed a reduction in phosphorylation of the players of the Ras pathway, including Ras, p-Raf1, and p- Erk1/2 proteins [26]. These findings suggest that aloperine can inhibit tumors by blocking the RAS pathway.

Bladder cancer is common and is a malignant type of cancer, occurring in the urinary system [158]. Despite the availability of treatment options, a quest for searching for relatively safe therapeutic candidates is going on. In one study, aloperine exhibited promising anti-tumor effects in bladder cancer cells. Zhang et al. treated EJ bladder cancer cells with aloperine and found out that treatment targeted Ras signaling to inhibit the growth of tumor cells. Aloperine downregulated the Ras protein and attenuated the phosphorylation of its effectors Raf1 and Erk1/2 [59]. These actions stopped the progression of the Ras/Raf1/Erk1/2 cascade and prevented the growth of bladder cancer cells (Figure 3).

**Table 2:** Aloperine mediated modulations in signaling mechanisms.

PI3K/Akt and Other Downstream Molecules Signaling						
Pathological Conditions	Cell Lines	Animal Model	Dosage		Regulatory Effects of Aloperine	Ref.
			In Vitro (µM)	In Vivo		
Prostate cancer	PC3, DU145 and LNCaP	BALB/C mice	100/200	30 mg/kg	Inhibition of Akt/ERK signaling	[22]
Hepatocellular carcinoma	Hep3B and Huh7	Zebrafish embryo	200/350/500	100 µM, 150 µM	Inhibition of PI3K/Akt signaling	[23]
Osteosarcoma	MG-63 and U2OS	---------	100/200	---------	Inhibition of PI3K/Akt signaling	[11]
Colon cancer	HCT116	---------	250/500	---------	Inhibition of PI3K/Akt signaling	[14]
I/R-Induced Renal Injury	RAW264.7 and HK2	C57BL/6 mice	500	50 mg/kg	Inhibition of PI3K/Akt/mTOR signaling	[15]
Thyroid Cancer	KMH-2 and IHH-4	---------	200	---------	Inhibition of Akt/mTOR signaling	[27]
Thyroid Cancer	IHH-4,8505c and KMH-2	--------	100/200	-------	Inhibition of Akt signaling	[30]
DSS-Induced Colitis	Jurkat Cells	C57BL/6 mice	250/500	40 mg/kg	Inhibition of PI3K/Akt/mTOR signaling	[29]
Microembolisation-Induced cardiac Injury	---------	Sprague-Dawley rats	---------	200 mg/kg	Activation of the PI3K/Akt signaling	[61]
I/R-Induced Cerebral injury	---------	Sprague-Dawley rats	---------	2/25/50 mg/kg	Activation of the PI3K/Akt signaling	[16]
**NF-κB Signaling**						
Allergic airway inflammation	---------	BALB/c mice	---------	100/200 mg/kg	Inhibition of NF-κB signaling	[18]
Neuropathic pain	---------	ICR mice	---------	80 mg/kg	Inhibition of NF-κB signaling	[31]
Intervertebral disc degeneration	Nucleus Pulposus cells	Sprague-Dawley rats	100	-------	Inhibition of NF-κB signaling	[44]
Pulmonary arterial hypertension	---------	Sprague-Dawley rats	---------	25/50/100 mg/kg	Inhibition of NF-κB signaling	[124]
Osteoporosis	RAW264.7	C57BL/6 mice	20	30 mg/Kg	Inhibition of NF-κB signaling	[121]
LPS-induced macrophage activation	RAW264.7	---------	50/100	---------	Inhibition of NF-κB signaling	[111]
**Nrf2/HO-1 Signaling**						
Allergic airway inflammation	---------	BALB/c mice	---------	100/200 mg/kg	Activation of Nrf2/HO-1 Signaling	[18]
Retinal pigment epithelial cells injury	ARPE-19	---------	6.25/12.5/25	---------	Activation of Nrf2/HO-1 Signaling	[19]
High Glucose induced Schwann cells injury	RSC96 cells	---------	1/10/50	---------	Activation of Nrf2/HO-1 Signaling	[21]
CCl4 induced mouse hepatic injury	---------	C57BL/6 mice	---------	50/100 mg/kg	Activation of Nrf2/HO-1 Signaling	[147]
**Ras Signaling**						
Breast cancer	MCF-7 and MDA-MB-231	---------	100/200/400	---------	Inhibition of Ras signaling	[26]
Bladder Cancer	EJ cells	----------	25/50/100	---------	Inhibition of Ras signaling	[59]

## 10. Conclusions and Future Prospects

Aloperine, an alkaloid from *Sophora alopecuroides* L., can produce therapeutic effects against multiple pathological conditions. For centuries, it has been used as Chinese traditional medicine to treat allergies and inflammatory conditions [8,159]. It has been extensively investigated for its remedial benefits against various diseases. Many studies revealed that aloperine could effectively improve abnormal conditions like chronic allergy, inflammation, pain, bacterial infections, viral infections, intervertebral disc degeneration, pulmonary fibrosis, and cerebral injury [7,8,12,13,14,15,60,159,160,161,162,163,164]. Aloperine also showed potent anticancer effects against cancers like osteosarcoma, prostate, lung, liver, thyroid, breast, and bladder cancer [11,87,165,166]. These outcomes revealed that aloperine produced therapeutic effects by activating or inhibiting vital biological processes like apoptosis, cell cycle, and autophagy. Furthermore, it has also been determined that aloperine is a potent regulator of important signaling mechanisms like PI3K-Akt-mTOR, NF-κB, Nrf2-HO1, and Ras. Aloperine could cause context-dependent modulations of these signaling mechanisms to achieve beneficial effects.

Many studies revealed that aloperine could trigger extrinsic and intrinsic apoptosis mechanisms to cause cell death in multiple diseases. Aloperine activated extrinsic apoptosis to improve pathological conditions like multiple myeloma, prostate cancer, and thyroid cancer [12,22,30]. Similarly, aloperine lessened the adverse effects of diseases like hepatocellular carcinoma, osteosarcoma, colon cancer, breast cancer, leukemia, alzheimer’s disease, non-small cell lung cancer, bladder cancer, and colorectal cancer by triggering intrinsic apoptosis pathway [7,11,14,23,24,26,40,43,59]. On the contrary, aloperine was also found to inhibit apoptosis. Oxidants and high levels of chemicals could trigger apoptosis, which promotes disease progression. Apoptosis could facilitate the occurrence of pathological conditions like IR-induced renal injury, intervertebral disc degeneration, OGD/RP neuronal injury, early brain injury, IR-induced cerebral injury, retinal pigment epithelial cells injury, DSS-Induced colitis, and microembolisation-induced cardiac injury [15,16,17,19,29,44,60,61]. Aloperine treatment caused inhibition of apoptosis to alleviate the detrimental effects of these abnormal conditions. These findings suggest that aloperine could produce context-dependent diversified apoptosis regulatory effects, but aloperine mediated inhibition or induction of apoptosis produced beneficial outcomes against diseases. However, it is needed to understand further the apparent reasons and underlying molecular mechanisms for the dual apoptosis regulatory role of aloperine in various cell types and diseases.

Aloperine could arrest the cell cycle at different phases to stop cell growth. Aloperine mediated cell cycle inhibitory effects are mainly found in various tumors. Aloperine caused G1 cell cycle arrest in prostate cancer and non-small cell lung cancer [22,24]. Aloperine also inhibited the cell cycle progression at the G2/M phase to suppress the growth of hepatocellular carcinoma, colon, and liver cancer [14,21,23]. Besides these promising effects, one study reported that aloperine could not affect the cell cycle progression in thyroid cancer, which revealed the tumor cell type-dependent cell cycle regulatory role of aloperine [30]. Aloperine was also reported to halt the migration and invasion of breast cancer, liver cancer, and bladder cancer cells [21,25,26], which proved that aloperine could inhibit the growth of the localized tumor and help prevent metastatic state disease.

Moreover, aloperine also modulated the autophagy process. It is well known that autophagy could facilitate or inhibit tumor cells growth depending on the state of the disease. Aloperine induced autophagy and produced cytotoxic effects in leukemia and KMH-2, IHH-4 thyroid cancer cells [7,27], but aloperine inhibited autophagic flux in 8505c thyroid cancer cells [27]. The aloperine triggered concomitant inhibition and induction of autophagy in thyroid cancer cells suggest that this dual role of aloperine should also be investigated, and its molecular mechanism should be deciphered in other cancer cell types.

The literature review exhibited that aloperine is an efficient modulator of vital signaling mechanisms, which control protein synthesis, cell proliferation, differentiation and help cells cope with stress conditions. Aloperine modulated the PI3K/Akt/mTOR pathway by upregulating or downregulating the levels of participants of this pathway. Aloperine treatment caused inhibition and activation of PI3K/Akt/mTOR to suppress multiple tumors’ growth and reduce the harmful effects of injurious conditions [11,14,15,16,22,27,30]. Aloperine also inhibited the NF-κB and Ras Signaling cascades and exerted anti-tumor and anti-nociceptive effects. Similarly, aloperine triggered inhibition of these pathways also contributed to eradicating disease-promoting immune and inflammatory responses [18,26,44,59,111,121]. Aloperine also proved to be an effective anti-oxidant. Oxidants are produced in the body mainly due to exposure to harmful chemicals or inflammation. Aloperine produced protective effects against the oxidants-related adverse conditions by activating the Nrf2/HO-1 Signaling cascade. Activating the Nrf2/HO-1 pathway by aloperine helped control the inflammatory and cell degrading conditions [19,32,147].

Pharmacokinetics (PK) explains how a drug is absorbed, distributed, metabolized, and cleared from the body after administration [167]. A literature review showed that, currently, limited data are available about the pharmacokinetics of aloperine. In one study, Huang et al. determined the pharmacokinetics of aloperine after administering aloperine 50 mg/kg orally and 5 mg/kg intravenously to male rats. The evaluation of pharmacokinetics parameters revealed T_1/2_ (half-life) 5.80 ± 1.09 h, T_max_ (time to reach maximum concentration) 0.96± 0.10 h, and the V_d_ (apparent volume of distribution) 69.44 ± 14.45 L/kg. These outcomes indicate rapid absorption and distribution of aloperine in animal tissues. Similarly, aloperine, being water-soluble, was efficiently excreted from the animal body and showed CL (Clearance) values of 8.33 ± 0.98 L/h/kg after oral, and 8.17 ± 1.11 L/h/kg after intravenous administration. Furthermore, aloperine showed 44.87% bioavailability in rat plasma samples [168]. These findings suggest that aloperine possesses acceptable pharmacokinetics behavior. However, there is a need to conduct extensive in vivo studies to further explore the pharmacokinetics of aloperine. The resulting data will help in drug development and employment of aloperine in clinical settings.

Above-mentioned findings prove aloperine to be a potent modulator of biological pathways. Aloperine has been extensively investigated in pre-clinical settings, and it produced promising disease eradicating outcomes and possesses encouraging PK behavior. There is a need to further explore the molecular mechanisms in different disease models and confirmation of already identified regulatory roles of aloperine, especially its dual regulatory role reported by several studies. An in-depth understanding of modulations of molecular mechanisms by aloperine could help its entry into clinical settings. Aloperine is a promising drug candidate, and it has the potential to produce broad-spectrum therapeutic effects against a variety of diseases.

## Data Availability

Not applicable.

## Abbreviations

AHR	Airway hyper-responsiveness
ALP	Alkaline phosphatase
ALT	Alanine transaminase
AMD	Age-related macular degeneration
Apaf-1	Protease activating factor-1
ARE	Antioxidant response element
AST	Aspartate aminotransferase
ATP	Adenosine triphosphate
BAFFR	B-cell activating factor receptor
Bax	Bcl2-associated X protein
Bcl2	B-cell lymphoma 2
BMM	Bone Marrow-Derived Macrophages
CCI	Chronic constriction injury (CCI)
CCl_4_	Carbon Tetrachloride
CD40	Cluster of differentiation 40
Cdc2	Cell-Division Cycle 2
Cdc25C	Cell division cycle 25
CDK	Cyclin-dependent protein kinase
cFLIP	Cellular FLICE (FADD-like IL-1β-converting enzyme)-inhibitory protein
CL	Clearance
CME	Coronary micro-embolization
CNC	Cap ‘n’ collar
CO	Carbon monoxide
DSS	Dextran sodium sulfate
E2F1	E2F Transcription Factor 1
EGF	Epidermal growth factor
EGFR	Epidermal growth factor receptor
EMT	Epithelial-mesenchymal transition
ERK	Extracellular signal-regulated kinase
ERK1/2	Extracellular signal-regulated kinases 1 and 2
FADD	Fas-associated death domain
GRO1	Growth Regulated Oncogene 1
GST	Glutathione S-transferase
GTP	guanosine 5’-triphosphate
H_2_O_2_	Hydrogen peroxide
HO-1	Heme oxygenase-1
IkBα	Nuclear factor of kappa light polypeptide gene enhancer in B-cells inhibitor, alpha
IKK	IκB kinase
IL-13	Interleukin-13
IL-4	Interleukin-4
IL-5	Interleukin-5
IL-6	Interleukin-6
IR	Ischemia and reperfusion
LC3	1A/1B-light chain 3
LTβR	Lymphotoxin beta receptor
MAPK	Mitogen-activated protein kinase
MCAO	Middle cerebral artery occlusion
MDA	Malondialdehyde
MEK	Mitogen-activated protein kinase kinase
MEK1/2	Mitogen-activated protein kinase kinases 1 and 2
MMP	Matrix metalloproteinases
mTOR	Mammalian target of rapamycin
NADPH	Nicotinamide adenine dinucleotide phosphate
NF-kB	Nuclear factor kappa-light-chain-enhancer of activated B cells
NIK	NF-κB-inducing kinase
NQO1	NAD(P)H: quinone oxidoreductase 1
Nrf2	Nuclear factor erythroid 2-related factor 2
NSCLC	Non-small cell lung cancer.
OGD-RP	Oxygen-glucose deprivation-reperfusion
PAH	Pulmonary arterial hypertension
PARP	Poly ADP ribose polymerase
PI	Propidium Iodide
PI3K	Phosphatidylinositol-3 kinase and PI3 kinase.
Rb	Retinoblastoma Tumor Suppressor Protein
PRR	Pattern recognition receptor
RANK	Receptor activator of nuclear factor κB
RANKL	Receptor activator of nuclear factor κB ligand
ROS	Reactive oxygen species
RPE	Retinal pigment epithelium
RTK	Receptor tyrosine kinase
SFDA	Chinese state food and drug administration
Snail	Zinc finger protein SNAI1
Th17	T helper cell 17
T_1/2_	Half-life
T_max_	Time to reach maximum concentration
TNFR	TNF receptors TNFR1
TNF-α	Tumor Necrosis Factor alpha
TRAIL	Tumor Necrosis Factor-Alpha-Related Apoptosis-Inducing Ligand
TRAIL-R1/2	Tumor Necrosis Factor-related Apoptosis-inducing Ligand Receptor 1/2
Tregs	Regulatory T-cells
Twist1	Twist-related protein 1
V_d_	Apparent volume of distribution
XAR	Xenobiotic-activated receptor (XAR)
